# A Novel Epigenetic Mechanism of Intestinal NHE3 Gene Regulation by Histone Acetylation

**DOI:** 10.3390/ijms27188096

**Published:** 2026-09-11

**Authors:** Anoop Kumar, Dulari Jayawardena, Shubha Priyamvada, Mitul Patel, Hayley Coffing, Neelkanth Kulkarni, Mumtaz Anwar, Arivarasu N. Anbazhagan, Pooja Malhotra, Ravinder K. Gill, Waddah A. Alrefai, Pradeep K. Dudeja, Seema Saksena

**Affiliations:** 1 Division of Gastroenterology and Hepatology, Department of Medicine, University of Illinois Chicago, Chicago, IL 60607, USA; 2 Jesse Brown VA Medical Center, 820 South Damen Avenue, Chicago, IL 60612, USA

**Keywords:** SLC9A3, ion transporter, IBD, HDAC inhibitor

## Abstract

NHE3 is an essential Na+ transporter in the intestine, contributing to diarrhea associated with inflammatory bowel disease (IBD) or intestinal infections. Nevertheless, the influence of histone acetylation on intestinal NHE3 gene expression has not yet been investigated. Various methods were employed, including human intestinal epithelial cells (Caco2 cells), ex vivo mouse and human ileal enteroids, and in vivo mouse models, along with techniques such as real-time qPCR, Western blot, immunofluorescence staining, ChIP, and H3K27Ac HiChIP sequencing analyses. Our findings indicated that valproic acid (VPA, a class I HDAC inhibitor) significantly elevated both NHE3 mRNA and protein levels in IECs. ChIP and Chromatin accessibility assays revealed an enhanced enrichment of acetylated histones (H3/H4) and a more open chromatin state at the p-1329/p-541 region of the NHE3 promoter in Caco2 cells treated with VPA. HDAC2/3 inhibition by MI192 in Caco2 cells and mouse/human ileal organoids or siRNA knockdown of HDAC2 in Caco2 cells significantly increased NHE3 expression. Activation or ectopic overexpression of histone acetylase p300 in Caco2 cells increased NHE3 mRNA expression and enhanced accumulation of acetylated histone (H3) within the p-1329 to -161 bp region of the NHE3 promoter. Additionally, in vivo experiments revealed that VPA significantly increased NHE3 mRNA levels in the ileum and proximal colon of mice, along with increased NHE3 immunostaining. H3K27Ac HiChIP sequencing analysis of isolated IECs from the mouse ileum indicated that VPA increased H3K27Ac acetylation and facilitated chromatin looping between distal enhancers and the NHE3 promoter, thereby stimulating NHE3 transcription. In conclusion, HDAC2 inhibition and histone acetylation play pivotal roles in upregulating NHE3 expression, with therapeutic implications for inflammation-associated diarrhea linked to reduced NHE3 expression/function.

## 1. Introduction

NHEs constitute a group of integral membrane proteins that facilitate the transport of extracellular Na+ into cells in exchange for intracellular H+. Among the 11 isoforms of NHE, four (NHE1–3, 8) are found in the intestine. NHE2, NHE3, and NHE8 are located in the apical membrane of intestinal epithelial cells (IECs), while NHE1 is situated on the basolateral side. NHE8 is present during early development and is expressed at lower levels in adulthood [1]. Studies involving gene knockout in mice have shown that NHE2 has little to no impact on intestinal sodium absorption, with NHE3 being the primary contributor [2,3]. Disruption of NHE3 expression and/or function leads to diarrhea and acid–base imbalance in mice [2,3,4]. Additionally, mutations in NHE3 have been associated with congenital sodium diarrhea (CSD) [4]. These findings highlight NHE3 as a promising therapeutic target for managing diarrhea associated with inflammatory bowel disease (IBD) and infections [1,5]. Consequently, understanding the mechanisms that regulate this essential intestinal transporter is vital. Recent research from our lab and others indicates that the expression and/or function of the NHE3 protein are rapidly adjusted through post-translational processes, including phosphorylation by cellular protein kinases or by changes in NHE3 surface levels [6]. Furthermore, earlier investigations by our team have revealed that transcriptional processes influence NHE3 regulation through changes in its gene expression [7,8,9]. In addition to the regulatory effects of transcriptional and post-transcriptional mechanisms mediated by miRNAs [10], our previous research has indicated that NHE3 gene expression is also influenced by epigenetic factors, including DNA methylation [11].

Previous research demonstrated that butyrate, recognized for its role as an HDAC inhibitor, enhanced the activity of the human NHE3 promoter and increased the binding of the transcription factors Sp1 and Sp3 to this promoter, through the dephosphorylation of Sp1/Sp3 by the serine/threonine phosphatases PP1 and PP2A [12]. Conversely, Keila et al. [13] demonstrated that butyrate increased the promoter activity of the rat NHE3 gene through the phosphorylation of Sp1 by a serine/threonine kinase (PKA). These findings further indicated that butyrate’s transcriptional impact on NHE3 gene expression is mediated by post-translational modifications (phosphorylation/dephosphorylation) of Sp1. Nevertheless, no studies to date have explored the role of histone acetylation on NHE3 gene expression. Thus, the present study aims to explore the mechanisms by which histone acetylation affects intestinal NHE3 gene expression.

In this context, alterations in the acetylation of histones bound to DNA are known to influence gene expression by modifying chromatin structure and altering transcription factor association [14,15]. Histone acetyl transferases (HATs) enhance gene transcription by attaching acetyl groups to lysine residues in core histones, resulting in active chromatin. On the other hand, histone deacetylases (HDACs) facilitate gene silencing by removing acetyl groups from lysine residues on core histones, thereby promoting chromatin condensation [16]. It is widely acknowledged that inhibiting HDACs leads to histone hyperacetylation, which in turn results in a more relaxed chromatin structure [15]. Notably, HDAC inhibitors have gained attention as promising options for treating inflammatory bowel diseases and cancers associated with colitis [17,18,19]. Oral administration of HDAC inhibitors like valproic acid (VPA, which inhibits class I and IIa HDACs 1, 2, 3, 4, 5, 7 & 8) or SAHA (suberoylanilide hydroxamic acid/vorinostat, a pan-HDAC inhibitor) has been demonstrated to alleviate disease severity in mice with DSS (dextran sulfate sodium) and TNBS (trinitrobenzene sulfonic acid)-induced colitis by lowering the levels of pro-inflammatory cytokines [17,20]. These results indicate that inhibiting HDACs might have positive effects in the treatment of gastrointestinal inflammatory disorders. However, the effects of HDAC inhibition on NHE3 gene expression in intestinal epithelial cells (IECs) remain poorly understood. Consequently, we investigated the impacts of HDAC inhibition on NHE3 gene expression using in vitro cell culture, enteroids (both mouse and human), and in vivo mouse models.

Our current studies have uncovered a new epigenetic mechanism that promotes NHE3 gene expression, involving histone acetylation, HDAC2 inhibition, and increased binding of acetylated histones (H3 or H4) to the NHE3 promoter. These findings imply that enhancing NHE3 expression could be a novel approach to counteract inflammation- and IBD-related diarrhea.

## 2. Results

### 2.1. Inhibition of HDAC by Valproic Acid Led to an Increase in NHE3 Gene Expression in Caco2 Cells

**Figure 1:**
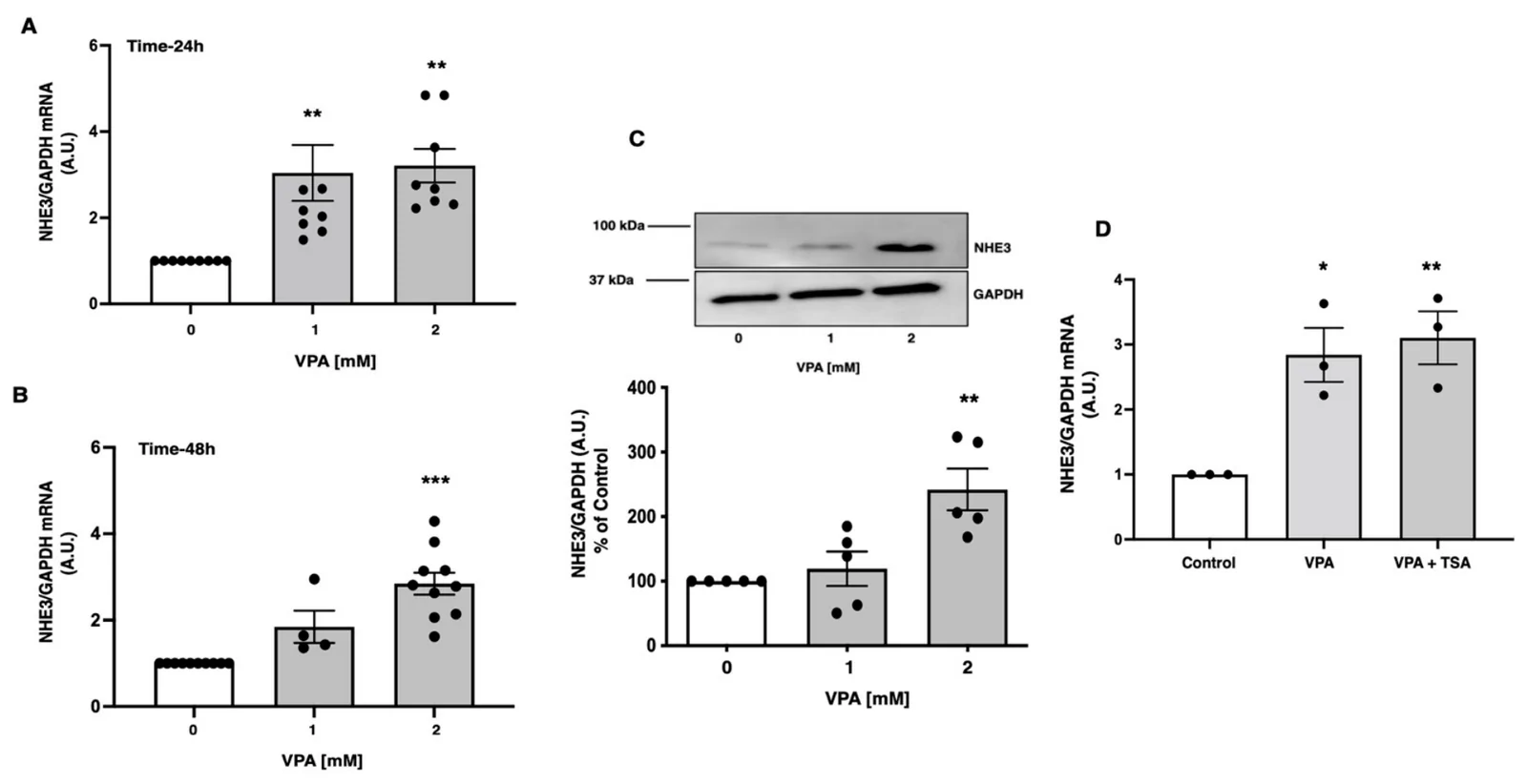
Valproic acid enhances NHE3 expression in Caco2 cells: Post-confluent Caco2 cells were exposed to VPA (1–2 mM) for 24 h (**A**) or 48 h (**B**), and the expression of NHE3 mRNA was evaluated using real-time qPCR. The expression of NHE3 protein was analyzed through immunoblotting following VPA treatment (24 h). A representative image of Western blot analysis, along with densitometric evaluation, is provided (**C**). Post-confluent Caco2 cells were treated with VPA (2 mM) alone or in combination with TSA (1 μM) for 24 h, and NHE3 mRNA expression was measured (**D**). The results are presented as fold increase in arbitrary units relative to the untreated control (set as 1 for mRNA or as a percentage of control for protein). The findings represent means ± SEM of 3–9 biological replicates. * *p* < 0.05, ** *p* < 0.01, *** *p* < 0.001 in comparison to untreated controls.

To examine the role of histone hyperacetylation in the regulation of NHE3 expression, Caco2 cells were treated with the well-known class I HDAC inhibitor, valproic acid (VPA, 1–2 mM) for 24 h or 48 h and NHE3 mRNA levels were assessed by real-time qPCR and protein expression by Western blotting. As shown in Figure 1A,B, the relative NHE3 mRNA expression increased significantly by approximately 2–3 times after treatment with VPA for 24 or 48 h in Caco2 cells. Simultaneously, VPA (2 mM, 24 h) also resulted in a significant rise in NHE3 protein levels in these cells (Figure 1C). It should be noted that in addition to class I HDACs (HDACs: 1, 2, 3, & 8), VPA also inhibits class IIa HDACs (HDACs: 4, 5 & 7) with an IC50 of ~1.5 mM but does not affect class IIb HDACs (HDAC6 and HDAC10) [21]. The IC50 of VPA against HDACs 1, 2, 3, 4, 5 & 7 ranges from 700–1500 µM, while the IC50 of VPA against HDAC6 & 8 ranges from 7442–20,000 µM [22]. Therefore, 1–2 mM VPA is unlikely to inhibit HDAC6 & 8. Moreover, we have previously shown that HDAC1 and HDAC2 are the predominant isoforms expressed in Caco-2 cells, while HDACs 3, 4, 5, 7, 8 & 9 are expressed at minimal levels [23]. Therefore, our data suggest that VPA-mediated inhibition of HDACs 1 & 2 increased NHE3 mRNA and protein expression in Caco2 cells. Interestingly, simultaneous addition of the broad-spectrum HDAC inhibitor trichostatin A (TSA, 1 µM) with VPA (2 mM; a class I & IIa HDAC inhibitor) did not show an additional synergistic increase in NHE3 mRNA levels (Figure 1D), implying that TSA and VPA stimulate NHE3 mRNA expression through a similar mechanism. Since the IC50 of TSA against HDAC4 & 7 ranges from 3.63–6.59 µM [22], our data further suggest that inhibition of HDAC4 or HDAC7 by 1 µM TSA does not increase NHE3 mRNA expression.

### 2.2. Valproic Acid Altered Histone Status at NHE3 Gene Promoter

**Figure 2:**
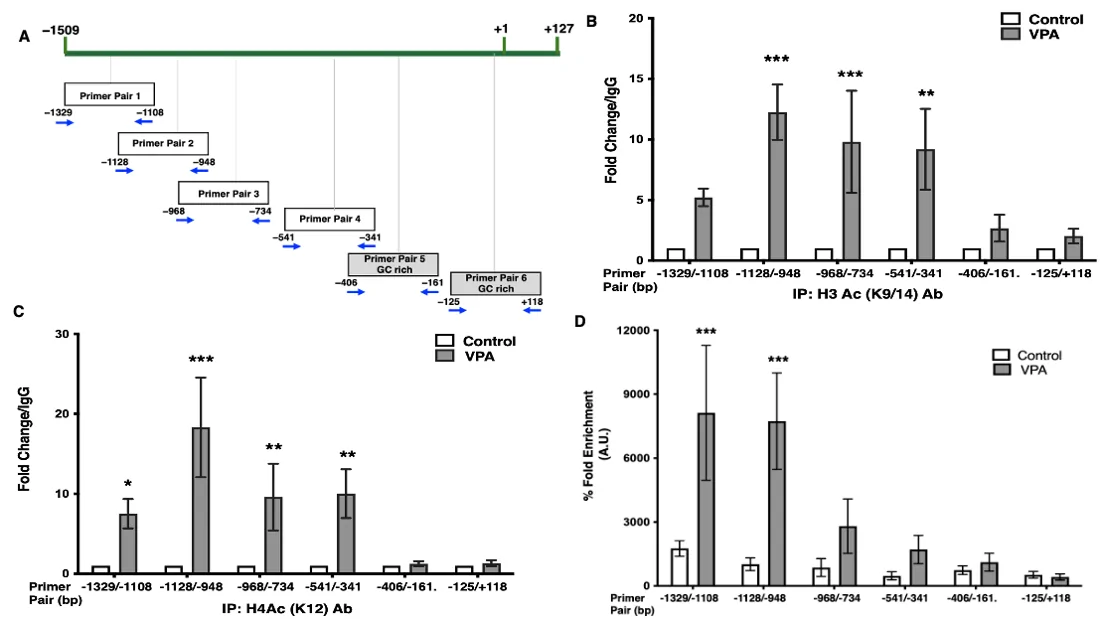
Enrichment of acetylated histones 3 and 4 (H3Ac, K9/14 and H4Ac, K12) at NHE3 promoter and associated chromatin states in response to valproic acid: (**A**). Primer pairs (1–6) that extend across the entire NHE3 promoter region (-1509/+127). (**B**,**C**). Post-confluent cells that were either untreated or treated with VPA (2 mM) were fixed in formaldehyde, chromatin was sonicated, and immunoprecipitated using specific antibodies against acetylated histone H3 or H4. Following reverse cross-linking and DNA extraction, the immunoprecipitated DNA served as the template for real-time qPCR with primer pairs (1–6) that span the complete length of the NHE3 promoter (p-1509/+127). The enrichment of acetylated histones H3 or H4 at the NHE3 promoter at various nucleotide positions across the full promoter region due to VPA is depicted. (**D**). **Chromatin accessibility:** To analyze the chromatin states of the NHE3 promoter in response to hyperacetylation induced by VPA, chromatin was extracted from control and VPA-treated post-confluent Caco2 cells, then subjected to digestion with a nuclease mix. The isolated DNA was then utilized as the template for real-time qPCR with primer pairs (1–6) that cover the entire NHE3 promoter region (-1509/+127) as outlined in the Materials and Methods section. Results represent means ± SEM of 4 biological replicates and are expressed as fold change over IgG (untreated control set as 1 for (**B**,**C**)) or % fold enrichment (indicating the degree of chromatin accessibility). * *p* < 0.05, ** *p* < 0.01, *** *p* < 0.001 compared with untreated control.

While VPA inhibits both class I and class IIa HDACs, the inhibitory activity of VPA is strongest against class I HDACs 1, 2, 3 [22]. Therefore, we next examined whether changes in histone proteins (particularly H3 and H4) contribute to the regulation of NHE3 gene expression. To determine if the acetylation of histone H3 at K9/14 lysine residues and the acetylation of histone H4 at K12 lysine residue correlate with the upregulation of NHE3 gene expression, we looked at the association between acetylated histones, H3 and H4, and the NHE3 promoter using ChIP assays. Chromatin from Caco2 cells, either untreated or treated with VPA (2 mM, 24 h), was immunoprecipitated with antibodies specific to acetylated histone H3 (K9/K14) or H4 (K12). The enrichment of acetylated histone H3 or H4 that immunoprecipitated with the NHE3 promoter was analyzed using real-time qPCR with specific primer pairs (1–6) that cover the entire length of the NHE3 promoter region (p-1509/+127) as depicted in Figure 2A. Our findings demonstrated a significant increase in acetylated histones H3 and H4 by 12-fold and 18-fold, respectively, in the region between p-1128 to -948 bp of the NHE3 promoter following VPA treatment. Additionally, regions extending from p-1329 to -1108 bp, p-968 to -734 bp, and p-541 to -341 bp of the NHE3 promoter exhibited an increase in acetylated histone H3 by 5-, 10-, and 9-fold, respectively. A similar increase in acetylated histone H4 was observed in the regions from p-1329 to -1108 bp, p-968 to -734 bp, and p-541 to -341 bp of the NHE3 promoter by 8- and 10-fold (Figure 2B,C). Conversely, in the region from p-406 to +118 bp of the NHE3 promoter, VPA only led to a ~3-fold increase in the accumulation of acetylated histone H3, and there was nearly no increase (by ~1.3-fold) in the accumulation of acetylated histone H4 in this region (Figure 2B,C). Since acetylation of H3 at lysine (K) 9, 14 and 27 residues and H4 at lysine (K)12 is associated with an increase in transcriptional activity [16], these results suggest that the acetylated histones in the region from p-1328 to -406 bp of the NHE3 promoter likely exist in an open chromatin configuration in response to VPA, which may significantly contribute to the increase in NHE3 gene expression observed.

#### Mapping of Chromatin States of Regions Spanning the Full Length of the NHE3 Promoter Region (p-1509/+127) by Chromatin Accessibility Assays

The accessibility of regulatory regions within chromatin is vital for various aspects of gene regulation. Nucleosomes located at regulatory sites restrict access of numerous regulatory and transcription factors to DNA, resulting in a closed chromatin state [24]. Conversely, the greater the accessibility of DNA (open chromatin state), the more likely adjacent genes are to be actively transcribed, thereby increasing gene expression [24]. Therefore, we conducted chromatin accessibility assays in untreated control cells and Caco2 cells treated with VPA (2 mM, 24 h) to determine the chromatin states of the regions encompassing the NHE3 gene promoter (p-1509/+127). Our findings indicated that the region from -1128 to -125 bp within the NHE3 promoter exhibited a partially open chromatin state under untreated control conditions. However, this partially open chromatin state transitioned to an open chromatin state in response to VPA-induced hyperacetylation (Figure 2D). We observed a significant increase in fold enrichment (which indicates the level of chromatin accessibility) in the segment between p-1329 and -948 bp of the NHE3 promoter, whereas the level of increase was more limited in the regions p-968/-734 bp and p-541/-341 bp of the NHE3 promoter. Consistent with our ChIP data, these findings suggested that the region from -1329 to -541 bp was in an open chromatin state due to VPA-induced histone acetylation. However, VPA did not alter the chromatin state in the region between p-406 and +118 bp, which remained partially open.

### 2.3. Inhibition of HDAC2 Resulted in an Increase in NHE3 Expression

**Figure 3:**
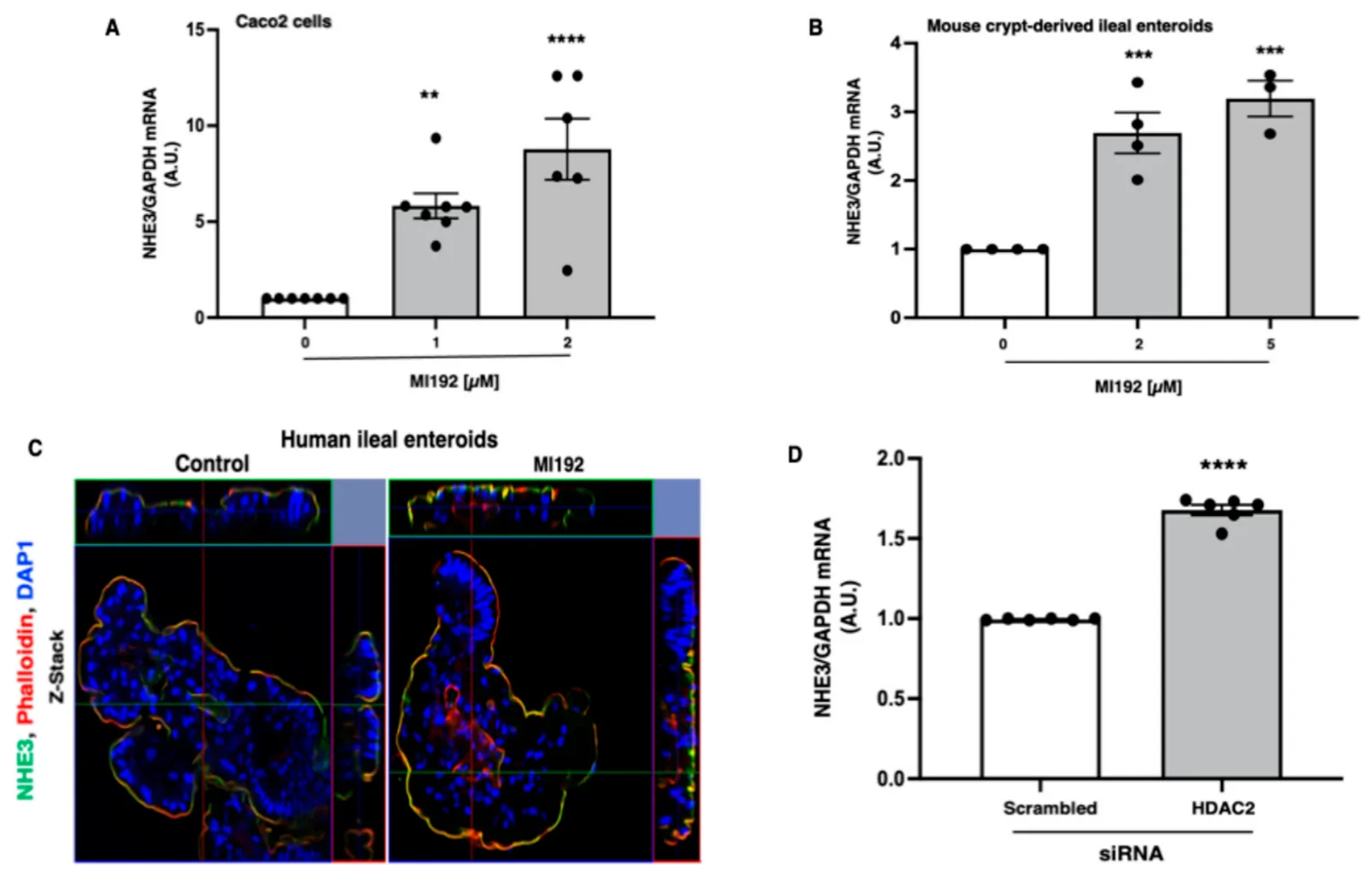
Inhibition of HDAC2 increases NHE3 expression: (**A**). Caco2 cells that had reached post-confluence were treated with the specific HDAC2/3 inhibitor, MI192 (1–2 μM), for 24 h. (**B**). Mouse ileal enteroids derived from crypts and cultured on Matrigel were treated with MI192 (2 or 5 μM) for 24 h. The expression of NHE3 mRNA was evaluated using real-time qPCR. The results are presented as a fold increase in arbitrary units relative to the untreated control (set at 1). The data represent means ± SEM from 3–7 biological replicates. ** *p* < 0.01, *** *p* < 0.001 in comparison to untreated Caco2 cells (control) or untreated enteroids (control). (**C**). Human ileal enteroids derived from crypts and maintained on Matrigel were treated with MI192 (2 M) for 24 h. Immunofluorescence staining illustrates NHE3 (green), phalloidin (red), and DAPI (blue, nuclei). A representative Z-stack confocal image was captured at 20× magnification. The stained enteroids were visualized with a Zeiss LSM 710 BiG confocal microscope (Zeiss, White Plains, New York, NY, USA) featuring a Plan-Apochromat 20× objective (Zeiss, White Plains, New York, NY, USA) and operated using ZEN 2.3 software. Scale bar: 20 µm. (**D**). Caco2 cells were transfected with either scrambled siRNA (control) or specific HDAC2 siRNA. The cells were harvested 72 h after transfection. The expression of NHE3 mRNA was evaluated through real-time qPCR. The results are shown as fold increase in arbitrary units relative to the untreated control (set to 1). The data indicate means ± SEM from 6 biological replicates. **** *p* < 0.0001 in comparison to cells treated with scrambled siRNA.

**(i) Caco2 Cells:** Since our results (Section 2.1) suggested that the increase in NHE3 mRNA and protein expression in response to VPA occurred via HDACs 1 and 2, we next investigated the direct impact of HDAC2 on NHE3 expression. The effects of the specific HDAC2/3 inhibitor, MI192, on NHE3 mRNA levels in Caco2 cells were assessed. It is important to note that MI192 does not affect HDAC1 [25]. Our findings revealed that, similar to the HDAC inhibition observed with VPA, MI192 at a concentration of 1–2 µM (for 24 h) led to an ~6 to 8-fold increase in NHE3 mRNA expression in Caco2 cells (Figure 3A), indicating that HDAC2 is likely the primary isoform responsible for mediating VPA effects on NHE3 mRNA expression. **(ii) Crypt-derived ileal enteroids:** To further support our findings in a more physiologically relevant context, mouse crypt-derived ileal enteroids were subjected to treatment with 2 or 5 µM of MI192 for 24 h. Consistent with the results obtained in Caco-2 cells, treatment with MI192 significantly increased (~3-fold) NHE3 mRNA expression in mouse ileal enteroids compared with untreated controls (Figure 3B). To enhance the translational relevance of our observations in mouse ileal enteroids, we also used human crypt-derived ileal enteroids for immunostaining. Notably, our data indicated that MI192 (2 µM, 24 h) elevated NHE3 protein levels in human crypt-derived ileal enteroids (Figure 3C), further supporting the role of HDAC2 in mediating VPA effects on NHE3 gene expression. **(iii) siRNA knockdown of HDAC2 in Caco2 cells:** Following 72 h of treatment with specific HDAC2 siRNA (100 nM), we observed a significant increase in NHE3 mRNA levels by ~50% (Figure 3D). These results confirm that specific knockdown of HDAC2 increased NHE3 expression.

### 2.4. NHE3 Expression Was Enhanced by Histone Acetylase, p300

**Figure 4:**
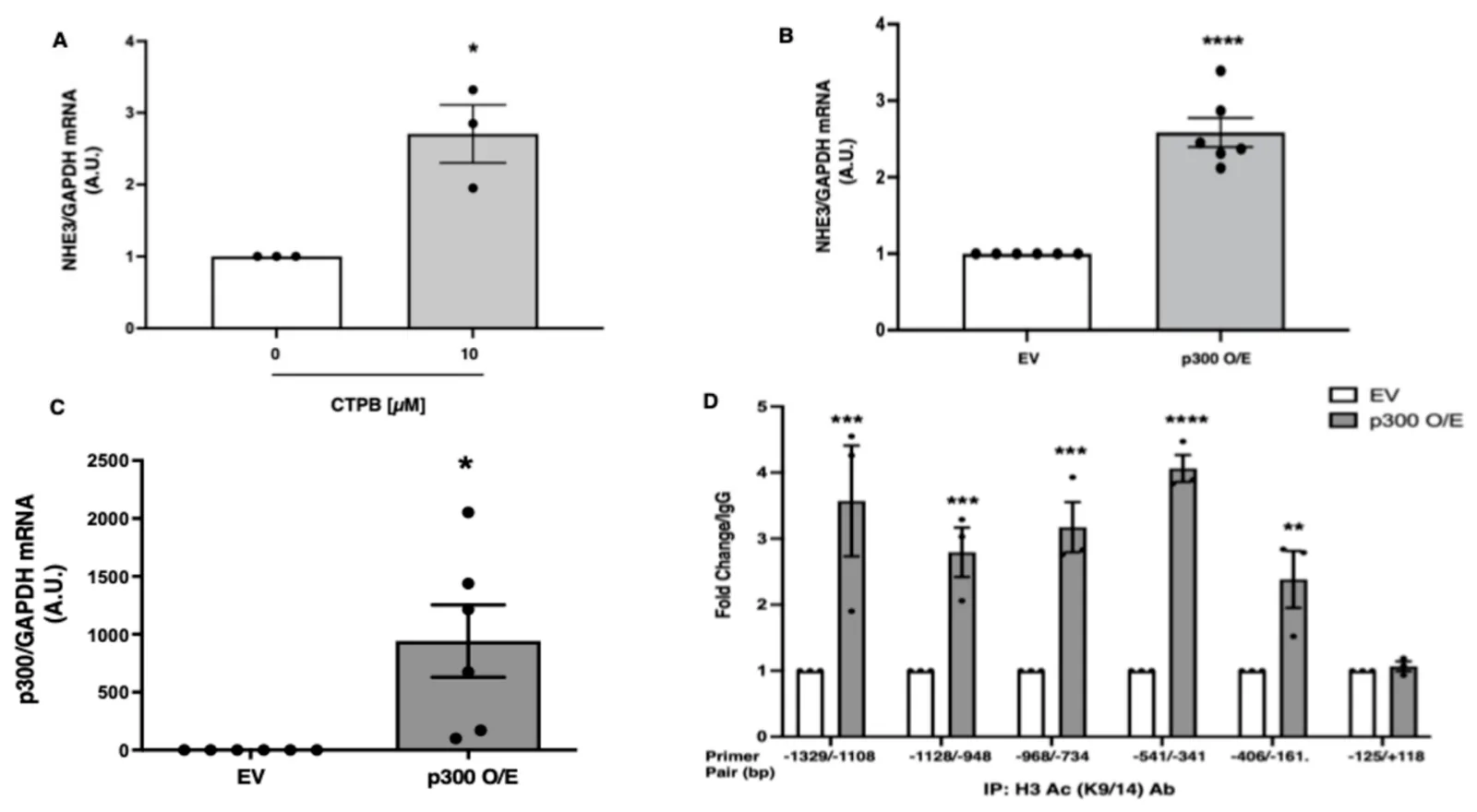
Histone acetylase p300 increases NHE3 expression: (**A**). Caco-2 cells after reaching confluence were treated with the p300 activator, CTPB (10 µM), for 24 h. NHE3 mRNA levels were measured using real-time qPCR. The results are reported as fold increase in arbitrary units relative to the untreated control (set to 1). Data represent means ± SEM from 3 biological replicates. * *p* < 0.05 in comparison to untreated cells (control). (**B**,**C**). Caco-2 cells were transiently transfected with either a p300 expression vector or an empty vector (pcDNA3.1). After 48 h of transfection, cells were collected, and mRNA levels of NHE3 and p300 were quantified by real-time qPCR. The results are reported as fold increase in arbitrary units relative to the empty vector (set to 1 for NHE3 mRNA). Data represent means ± SEM from 6 biological replicates. * *p* < 0.05, **** *p* < 0.0001 when compared to the empty vector (EV). (**D**). **ChIP assays:** Caco-2 cells transiently transfected with either the p300 expression vector or the empty vector (pcDNA3.1) were fixed in formaldehyde, followed by chromatin sonication and immunoprecipitation using an antibody against acetylated histone H3. Following reverse cross-linking and DNA extraction, the immunoprecipitated DNA served as the template for real-time qPCR, with primer pairs covering the entire NHE3 promoter (p-1509/+127). The enrichment of acetylated histone H3 associated with the NHE3 promoter at various nucleotide positions throughout the full promoter length induced by VPA is illustrated. The results represent means ± SEM from 3 biological replicates and are expressed as fold changes relative to IgG (with the empty vector set to 1). ** *p* < 0.01, *** *p* < 0.001, **** *p* < 0.0001 in comparison to the empty vector (EV).

The histone acetylase (HAT) p300 has demonstrated intrinsic histone acetyltransferase activity [26] and interacts with various transcription factors such as Sp1, Sp3, and HNF4α [27,28], positively influencing their transactivation efficiency. We then investigated the impact of HAT p300 on NHE3 expression in Caco2 cells. Using the p300 activator CTPB [29] at 10 µM for 24 h, our findings indicated a nearly 2-fold increase in NHE3 mRNA expression in Caco2 cells (Figure 4A). We also assessed whether the overexpression of p300 in Caco-2 cells affects NHE3 mRNA levels. Cells were transfected with either the p300 cDNA expression vector or an empty pcDNA3.1 vector. The relative levels of NHE3 mRNA were subsequently measured by real-time qPCR. The overexpression of p300 in Caco2 cells led to an increase in NHE3 mRNA expression by ~2.5-fold (Figure 4B). The transfection of Caco2 cells with the p300 expression vector was confirmed by real-time qPCR using primers specific to p300 (Figure 4C). In addition, ChIP assays were conducted to investigate the association of acetylated histone 3 (H3K9/14) with the NHE3 promoter in Caco2 cells that were transfected with the p300 cDNA expression vector or the empty pcDNA3.1 vector using specific antibodies against acetylated histone 3 (H3K9/14) or control IgG. Our results revealed a greater accumulation of acetylated histone H3 in the region from -1329 to -161 bp of the NHE3 promoter in Caco2 cells transfected with p300 compared to the empty vector (EV). However, we noted that there was virtually no effect on the enrichment of acetylated histone H3 within the region from -125 to +118 bp of the NHE3 promoter in p300-transfected Caco2 cells (Figure 4D). In summary, our results indicated that p300 histone acetylase activity leads to histone hyperacetylation and subsequent activation of NHE3 expression.

### 2.5. Valproic Acid Increased NHE3 Expression in the Mouse Intestine

**Figure 5:**
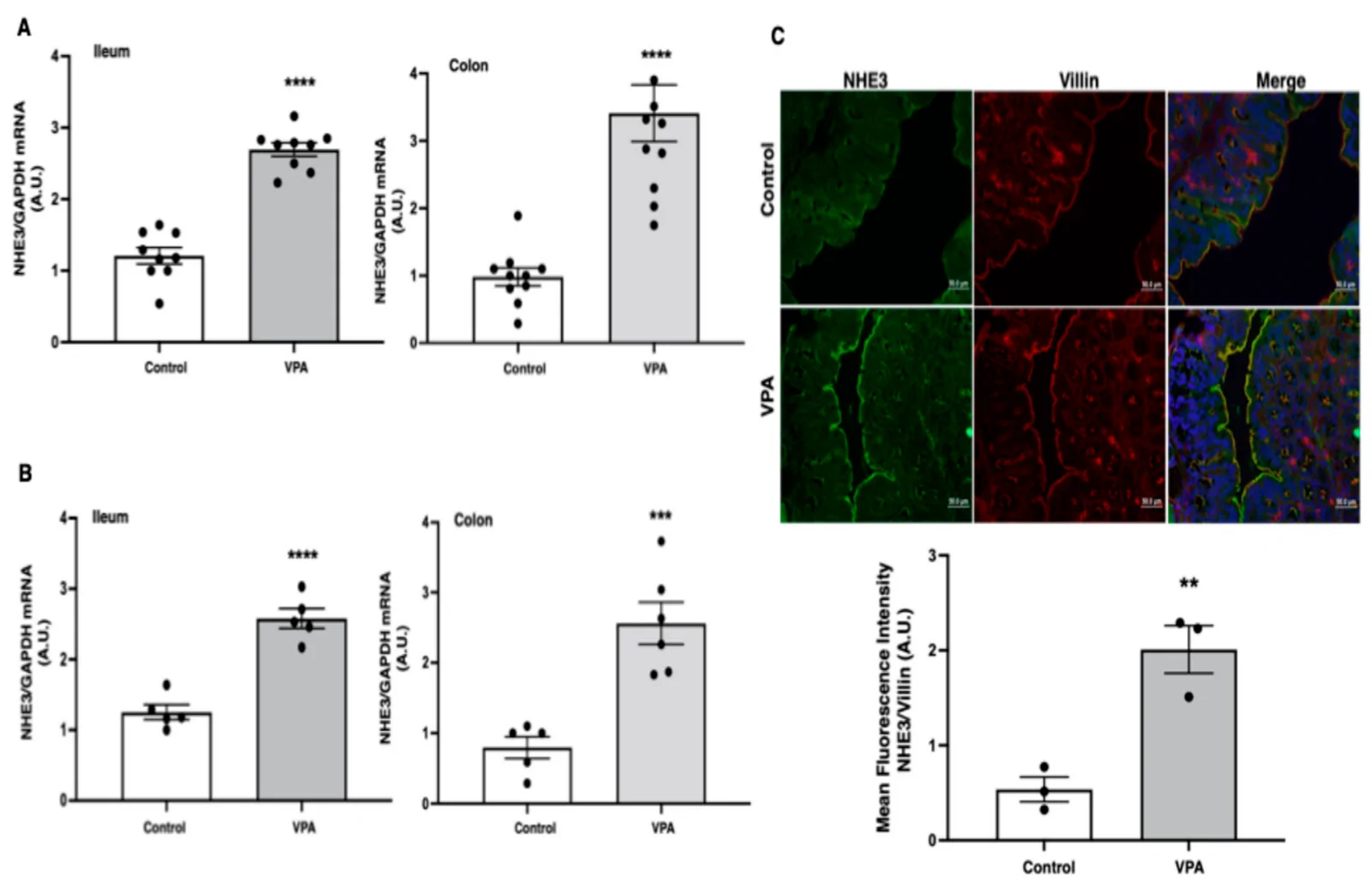
Valproic acid increases NHE3 expression in mouse ileum and colon: Valproic acid (VPA) was given to mice through intraperitoneal injections (once daily for 3 days) at dosages of 150 mg/kg body weight (**A**) or 400 mg/kg body weight (**B**), after which the ileum and colon were collected. Total RNA was isolated from the mucosal scrapings of the ileum and colon, and NHE3 mRNA expression was measured using real-time qPCR. The relative levels of NHE3 mRNA were normalized to GAPDH mRNA (the internal control). The results are presented as fold increases in arbitrary units in comparison to untreated controls. Data are expressed as means ± SEM for 5–10 mice per group. *** *p* < 0.001, **** *p* < 0.0001 compared to untreated mice (control). (**C**). A representative image showing the immunostaining of NHE3 protein in the proximal colon of both untreated (control) and VPA-treated mice. NHE3 is shown in green, villin in red, and DAPI (blue, indicating nuclei). The stained sections were observed using a Zeiss Axio Observer 5 inverted fluorescence microscope (Zeiss, White Plains, New York, NY, USA) equipped with an Axiocam 702 camera (Zeiss, White Plains, New York, NY, USA), set to 20× magnification. The scale bar measures 50 μm. The quantification of NHE3/villin as an indicator of average fluorescence intensity is provided. Results are shown as means ± SEM from 3 mice per group. ** *p* < 0.01 compared with untreated mice (control).

Consistent with our in vitro findings, the in vivo experiments in mice demonstrated that VPA (150 mg per kg body weight, administered intraperitoneally once daily for three days) [20] significantly elevated NHE3 expression by approximately 2.5 times in the ileum and colon (Figure 5A) compared to untreated control mice. A similar rise in NHE3 mRNA levels in the ileum and colon was also found at a dosage of 400 mg/kg body weight (Figure 5B). However, there were no changes in NHE2 mRNA levels in the ileum and colon following VPA treatment (Not displayed), indicating that the effects of VPA were specific to NHE3. Immunostaining analyses also revealed increased NHE3 protein levels in the proximal colon of VPA-treated mice compared with untreated control mice (Figure 5C). These results support the role of histone acetylation in regulating intestinal NHE3 expression in vivo.

### 2.6. Profiling of Regulatory Elements (Enhancers) at the NHE3 Gene Locus in Response to Valproic Acid

**Figure 6:**
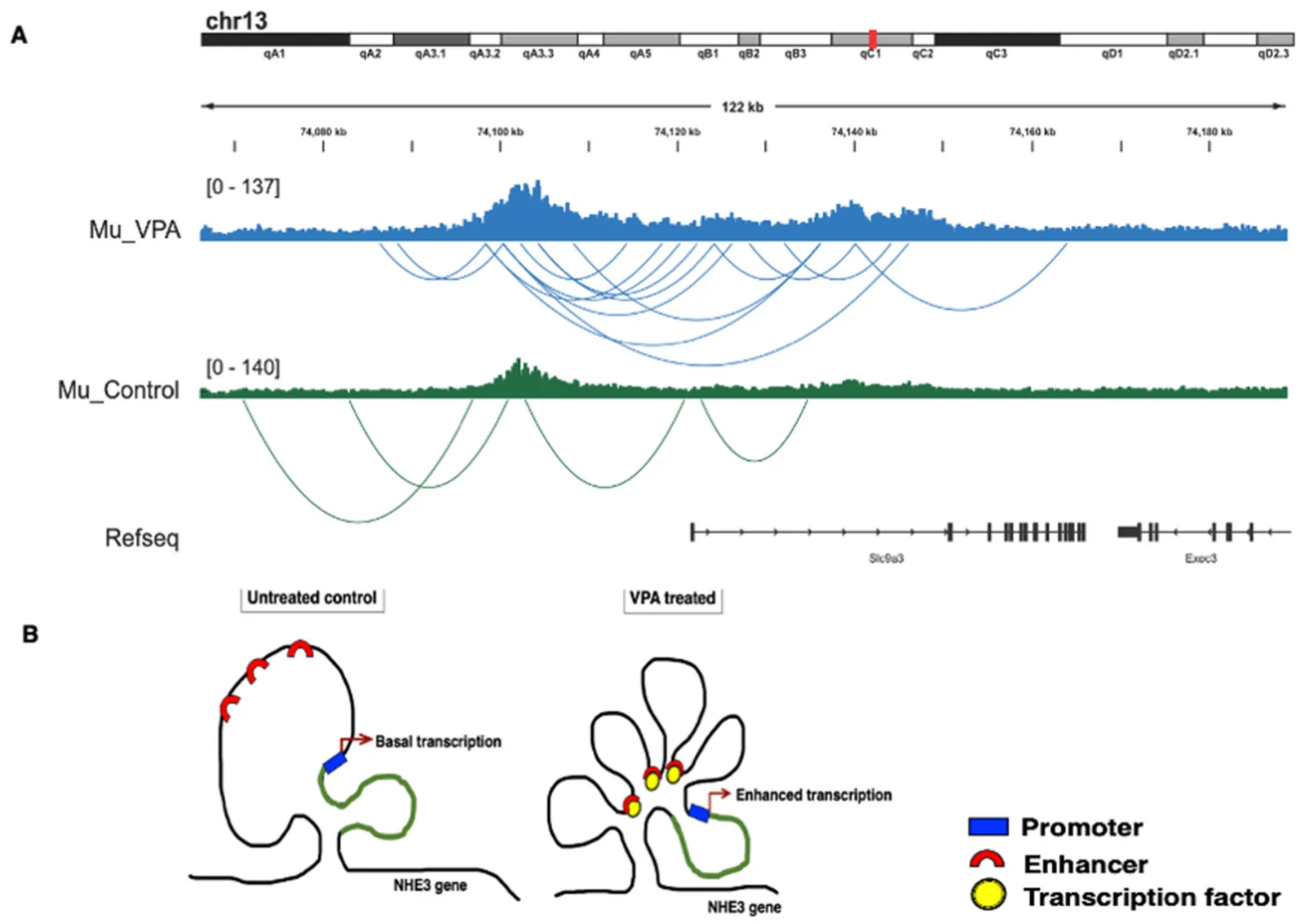
Enhanced histone acetylation and increased enhancer interaction frequency at NHE3 (slc9a3) gene locus in response to VPA: (**A**) Coverage profiles for H3K27ac were generated for both VPA-treated (blue) and untreated control (green) samples across a 122 kb genomic region encompassing Slc9a3 on chromosome 13. Epithelial cells were isolated from the ileum of untreated control (3 mice pooled as one sample) or VPA-treated mice (4 mice pooled as one sample). Contact frequency was defined as the number of HiChIP read pairs linking two genomic loci (as outlined in the Section 4). Contacts with frequencies exceeding the sample-specific mean contact frequency plus one standard deviation (mean + 1 SD) were retained and depicted as arcs. This criterion served as a visualization threshold for higher-frequency contacts and does not signify a formal statistical significance test of individual contacts or differential interactions between VPA-treated and control samples. The height of the arcs is scaled according to the genomic distance between the interacting loci and does not represent the strength of the interactions. The RefSeq track indicates the genomic positions of Slc9a3 and its neighboring genes. (**B**) A proposed model illustrating the interaction among the enhancer, transcription factor (TF), and the NHE3 (slc9a3) promoter, leading to increased NHE3 (slc9a3) transcription in response to VPA.

We performed H3K27Ac HiChIP sequencing analysis in intestinal epithelial cells (IECs) obtained from the ileum of both untreated (control) and VPA-treated mice to assess regulatory elements (enhancers) at the NHE3 (slc9a3) gene locus. Prior to conducting H3K27Ac HiChIP sequencing analysis, we evaluated the impact of VPA (400 mg/kg body weight, administered intraperitoneally once daily for 3 days) on NHE3 mRNA and protein levels in isolated mouse ileal IECs. As illustrated in Supplementary Figure S1, our results indicated that VPA increased NHE3 mRNA and protein expression in isolated ileal IECs, consistent with the effects observed in ileal and colonic mucosa (Figure 5B). The H3K27Ac HiChIP sequencing indicated that for the untreated control (illustrated in green), H3K27Ac acetylation marks, which are indicative of enhancers, were present within the approximately 74,100 kb region (green peaks). In contrast, the VPA treatment demonstrated significantly increased and enriched H3K27Ac acetylation marks (blue peaks) within the ~74,100 kb to ~74,140 kb region compared to the untreated control (Figure 6A).

Furthermore, untreated control ileal IECs (green) exhibited very few and shorter contact arcs between the enhancers and the NHE3 (slc9a3) promoter (marked in red, located within the ~74,140 kb region), whereas we observed a greater number of taller contact arcs in VPA-treated (blue) ileal IECs between the enriched acetylated enhancer regions and the NHE3 (slc9a3) promoter. These arcs represent interactions (chromatin loops) between genomic regions. The heights of these contact arcs correspond to the distance of interactions between the enhancer and the NHE3 (slc9a3) promoter, with such regulatory interactions being possible over distances greater than approximately ~10 kb upstream or downstream of the transcriptional start site of the gene promoter [30,31]. Figure 6B depicts a schematic representation of Chromatin looping. Chromatin looping, which brings potential enhancer regions containing transcription factors located far from the gene promoter closer to the gene promoter to facilitate transcription, may be a key mechanism underlying VPA-induced enhancement of NHE3 transcription in ileal IECs.

## 3. Discussion

Histone acetylation is a crucial epigenetic process that influences chromatin structure, DNA accessibility to transcription factors, and gene expression [16,24]. Our latest findings provide new insights into how histone acetylation affects NHE3 expression in intestinal epithelial cells (IECs). The process of histone acetylation is modulated by two types of enzymes: histone acetyltransferases, which add acetyl groups to the lysine residues of histones, and HDACs, which facilitate the removal of acetyl groups from either lysine residues of histones or specific transcription factors [16]. Our results indicated that inhibition of HDACs 1, 2, 3, 4, 5 & 7 [22] with valproic acid (VPA) increased both NHE3 mRNA and protein levels in the human intestinal model, Caco-2 cells. The impact of valproic acid (VPA) appeared to be specific to NHE3, as it did not affect the mRNA levels of another apical NHE isoform, NHE2. Moreover, when Caco-2 cells were treated simultaneously with VPA and trichostatin A (TSA, broad-spectrum HDAC inhibitor), there was no further increase in NHE3 mRNA levels, indicating that the enhancing effects of VPA and TSA on NHE3 mRNA likely occurred through a similar mechanism.

Studies have demonstrated that alterations in gene expression coincide with chromatin remodeling, which affects chromatin folding and function [16,24]. This chromatin remodeling is initiated by modifications such as acetylation, methylation, and phosphorylation of the histone tails [16,24]. Among the various histone modifications that have been identified, the acetylation of histone H3 at lysines (K) 9 and 14, and histone H4 at lysine (K) 12, along with the methylation of histone H3 at lysine (K) 4, are the most thoroughly studied, as the regulation of these modifications is associated with transcriptional activation [32]. It is important to note that VPA has been shown to promote histone acetylation, particularly of histones H3 and H4 [33], and that the VPA-induced hyperacetylation of these histones influences epigenetic marks, leading to chromatin decondensation and the activation of various promoters [33]. Notably, previous research indicated that butyrate, another well-known HDAC inhibitor [34] enhanced NHE3 gene promoter activity through a phosphatase-mediated dephosphorylation of the transcription factors Sp1 and Sp3, as well as increasing Sp1/Sp3 binding to the NHE3 promoter [12]. However, those studies did not examine the impact of histone acetylation on butyrate-mediated stimulation of the NHE3 gene promoter. In this study, we evaluated the influence of histone acetylation on NHE3 gene expression regulation in intestinal epithelial cells (IECs) by VPA. Our findings indicated that VPA increased the levels of acetylated histone H3 (K9/14) and H4 (K12) on the NHE3 gene by approximately 5–11-fold in the region extending from -1328 to -541 bp. These results further suggested that acetylation of histones H3 and H4 within the p-1328 to -541 bp range promotes the formation of more open, relaxed chromatin, thereby increasing NHE3 gene expression. In contrast, we observed a 3-fold increase in the accumulation of acetylated histone H3, along with a minimal increase (1.3-fold) in the accumulation of acetylated histone H4 by VPA within the p-406 to +118 bp region of the NHE3 promoter. Interestingly, the chromatin in this area was partially open.

Interestingly, this area (p-406 to +118 bp) has been identified as containing a CpG Island (CGIs; -353/+70 bp). We have previously shown that this region of the NHE3 promoter was methylated and that methylation was significantly reduced by the DNA methyltransferase (DMT) inhibitor 5-Azacytidine (5-Aza) in Caco2 cells [11]. In addition, mice treated with 5-Aza exhibited increased NHE3 expression in the ileum and colon [11]. Therefore, the partially open chromatin state in the p-406 to +118 bp segment of the NHE3 promoter seems to be linked to DNA methylation, with VPA treatment alone proving inadequate to promote histone acetylation in this region. In this context, studies have highlighted how DNA methylation affects chromatin state and organization at CpG island (CGI) regions, leading to a heterochromatin state [35]. It is probable that the impact of DNA methylation on chromatin structure within the p-406 to +118 bp region of the NHE3 promoter may operate through methyl CpG-binding proteins (MBD), which recruit co-repressor complexes associated with HDAC activity [36]. Nevertheless, the role of methyl CpG-binding proteins in promoting a partially open chromatin state within the p-406 to +118 bp region of the NHE3 promoter remains unexplored and will be the focus of future research.

Additionally, our findings indicate that inhibiting HDACs 1 and 2 may mediate VPA’s stimulatory effects on NHE3 expression in Caco2 cells, a finding supported by previous studies [21,22] and our own studies [23]. Furthermore, studies with the HDAC2/3 inhibitor, MI192, supported the role of HDAC2 inhibition in mediating VPA effects on NHE3 expression, as MI192 does not inhibit HDAC1 [25] and HDAC3 expression is low in Caco2 cells [23]. Our inhibitor studies were further confirmed using specific HDAC2 siRNA, which showed that HDAC2 knockdown in Caco2 cells increased NHE3 expression. MI192 also increased the NHE3 mRNA expression in ileal enteroids derived from crypts in both mice and humans. Considering that HDACs 1, 2, and 3 are notably expressed in the intestinal epithelium of the small intestine in both species [37,38,39,40], our results suggest that inhibiting HDAC2 and/or HDAC3 may play a role in the effects of VPA on NHE3 gene expression in the ileum of both mice and humans. Our results, suggesting that histone acetylation is crucial in regulating NHE3 gene expression in intestinal epithelial cells, were further validated using CTPB, a specific activator of p300 histone acetylase and its target genes [41,42]. We showed that CTPB significantly increased NHE3 expression in Caco2 cells. A similar increase in endogenous NHE3 mRNA levels was observed in Caco2 cells ectopically overexpressed with p300. p300, classified as a nuclear (type A) HAT, is known to acetylate histones that are already organized into chromatin, favoring histones H3 and H4 [43]. Interestingly, our findings indicated that overexpression of p300 in Caco2 cells increased the accumulation of acetylated histone H3 in the region from -1329 to -161 bp of the NHE3 promoter, whereas no effect was observed on the enrichment of acetylated histone H3 in the region from -125 to +118 bp of the NHE3 promoter. This suggests that the presence of a portion of the CpG island (p-353/+70 bp) in the -125 to +118 bp region of the NHE3 promoter may be influencing DNA methylation, which could affect the acetylation status of histone H3 and consequently alter the chromatin state of the -125 to +118 bp region of the NHE3 promoter. Therefore, these studies suggest that histone acetylation via activation of the histone acetyltransferase p300 and inhibition of histone deacetylase 2 (HDAC2) is essential for promoting NHE3 expression in intestinal epithelial cells (IECs).

We further validated our in vitro results in Caco2 cells by utilizing an in vivo mouse model, which more accurately reflects the complex characteristics of the natural intestine under normal physiological conditions. The administration of valproic acid (VPA) in mice led to a significant increase in NHE3 expression in both the ileum and colon, with no effect on NHE2 expression, suggesting that histone acetylation is not universal. Our results indicate that increased NHE3 expression in the intestine is attributed to histone acetylation through HDAC inhibition. A limitation of this study is the absence of functional Na+ transport activity in response to VPA; hence, in the absence of functional data, the physiological significance is only partially validated. Recent studies have demonstrated that, in addition to its role in Na+/H+ transport, NHE3 also regulates epithelial proliferation, migration, cell–ECM interactions, and the wound-healing process, underscoring its non-canonical functions in the intestine [44]. The role of histone acetylation in modulating the non-canonical functions of intestinal NHE3 is still a topic for future research. It is essential to understand that VPA influences gene expression through various secondary mechanisms and off-target effects that operate independently of HDAC inhibition. VPA can reduce DNMT1 (DNA methyltransferase 1) expression, facilitating passive DNA demethylation and reactivating genes previously silenced by promoter hypermethylation [45]. Moreover, VPA has been shown to inhibit the deacetylation of the non-histone protein p53 [46]. In addition, VPA can affect non-epigenetic pathways, such as the MAPK/ERK and Wnt/β-catenin signaling pathways, which, in turn, govern the activation and positioning of upstream transcription factors at specific gene promoters, resulting in the expression of target genes [47,48]. However, our HDAC2/3 inhibitor, MI192, and HDAC2 siRNA studies convincingly demonstrated that the observed increase in NHE3 expression in intestinal epithelial Caco2 cells, mouse, and human ileal enteroids results exclusively from histone acetylation.

The use of HDAC inhibitors has been shown to reduce intestinal inflammation [17,18,19]. Earlier studies demonstrated that the HDAC inhibitors SAHA and VPA reduced inflammatory alterations in DSS- or TNBS-induced colitis by minimizing histological evidence of inflammation, lowering levels of pro-inflammatory cytokines and chemokines, and increasing histone 3 acetylation [17,20]. In fact, intestinal epithelial cells at active sites of ulcerative colitis (UC) or Crohn’s disease (CD) displayed significantly lowered levels of histone H3 acetylation in comparison to healthy controls, suggesting increased HDAC activity [49,50]. Enhanced HDAC activity was also identified in the inflamed colonic epithelium of DSS-treated mice [50]. Furthermore, HDAC inhibitors such as SAHA and ITF2357 (Givinostat) were found to prevent TNFα-induced disruption of epithelial barrier integrity by promoting the expression of tight junction proteins, occludin and claudin-1, while reducing claudin-2 in T84 and murine CMT93 cells [51]. Overall, these studies suggest that HDAC inhibitors have the potential to alleviate intestinal inflammation by modulating key signaling pathways, improving intestinal barrier function, and facilitating immune regulation. Nonetheless, many HDAC inhibitors act on multiple HDACs in a nonspecific manner, which can lead to reduced precision and significant adverse effects in clinical use, including thrombocytopenia, fatigue, and diarrhea [52]. These side effects pose potential challenges for the use of pan-HDAC inhibitors in patients with IBD. On the other hand, employing inhibitors that specifically target individual HDAC isoforms may lessen systemic side effects by minimizing the disruption of other HDAC functions. In this context, MS-275 (Entinostat), which primarily inhibits HDAC1 and HDAC3 activity, has been shown to reverse inflammation associated (i) reduction in acetylation, (ii) activation of NF-κB, and (iii) inhibition of the tight-junction proteins zonula occludens 1 (ZO-1) and occludin in mice with DSS-induced colitis [17]. Also, combination therapies pairing HDAC inhibitors with current IBD treatments, such as biologics and small-molecule drugs, can improve overall effectiveness and patient outcomes. In this regard, an active clinical trial (NCT03167437) is assessing the effectiveness of vorinostat in combination with ustekinumab for managing moderate-to-severe Crohn’s disease (CD) and is currently in Phase 2 [52]. At present, clinical data are limited, underscoring the urgent need for more suitable human clinical trials.

In addition to their ability to reduce inflammation, our current studies have prompted us to propose that enhancing the expression of NHE3 via HDAC inhibition (which leads to increased histone acetylation) could be a promising strategy to alleviate diarrhea linked to inflammation, such as diarrhea associated with IBD, where both the expression and function of NHE3 are reduced. Further studies are needed to investigate changes in histone acetylation at the NHE3 promoter and to determine whether NHE3 expression can be restored with HDAC2 inhibition during inflammatory conditions in the intestine. These findings will definitively establish an essential mechanistic link between alterations in histone acetylation at the NHE3 gene and IBD-related diarrhea.

We aimed to more closely link the role of VPA to the active enhancer-chromatin state and chromatin interactions at the NHE3 gene promoter in ileal epithelial cells using H3K27Ac HiChIP sequencing. Given that genomes are organized in a three-dimensional arrangement rather than a linear fashion, chromatin conformation capture offers insights into the transcriptional landscape of chromatin in 3D and allows us to analyze chromatin folding characteristics associated with regulatory elements (e.g., enhancers and transcription factors) and their engagement with gene promoters. Transcriptional enhancers are segments of DNA that are marked by acetylated histones at H3 lysine 27 (H3K27). Enhancers boost transcription of adjacent genes, regardless of orientation, by looping over substantial genomic distances to activate gene promoters [53]. We observed increased H3K27 acetylation signals (indicative of active chromatin marks/enhancer regions) correlated with increased chromatin loops (enhancer-NHE3/slc9a3 promoter interactions) at the NHE3 (slc9a3) gene locus in ileal IECs from VPA-treated mice in comparison to untreated controls, suggesting that potential enhancer regions are in close proximity to the NHE3 gene promoter, leading to increased transcriptional activation of NHE3. Notably, the nucleotide sequence of human NHE3 exhibits 82% identity with that of rodent NHE3 [54]. Consequently, the H3K27Ac HiChIP sequencing approach revealed a mechanism through which VPA facilitates the opening of accessible chromatin regions and promotes 3D chromatin looping between enhancer regions and the NHE3 (slc9a3) gene promoter, thereby activating NHE3 transcription (Figure 6B). Our future goal will be to analyze the interacting regions and identify regulatory elements/enhancer regions that contain potential transcription factor binding sites, which may significantly influence the transcriptional activation of NHE3 in response to VPA.

In conclusion, our results demonstrate novel regulatory mechanisms for upregulation of intestinal NHE3 gene expression via epigenetic changes (Graphical Abstract), involving histone acetylation/HDAC2 inhibition, increased association of acetylated histones H3 and H4 with the NHE3 promoter, and enhanced enhancer-promoter chromatin looping. These novel mechanisms of NHE3 upregulation may provide a basis for new, more efficacious therapeutic modalities for intestinal disorders such as IBD-associated diarrhea, in which NHE3 expression/function is downregulated.

## 4. Materials and Methods

The chemicals and antibodies used in the experiments are described in Table 1. All other chemicals were of at least reagent grade and were obtained from either Sigma Chemicals Co. (St. Louis, MO, USA) or Fisher Scientific (Pittsburgh, PA, USA).

**Cell culture and treatment:** Human intestinal Caco-2 cells (catalog#HTB-37, ATCC, Manassas, VA, USA) were grown routinely in T-75-cm^2^ plastic flasks at 37 °C in a 5% CO_2_, 95% air environment as described previously [8,11,55]. Caco2 cells were plated at a density of 2 or 4 × 10^4^ cells/well on 12- or 6-well regular or Transwell plates for 12–14 days. Fully differentiated Caco-2 monolayers were treated with valproic acid (VPA; 1–2 mM) or trichostatin (TSA; 1 μM) for 24 or 48 h from the apical side in the serum-free cell culture medium supplemented with 0.2% bovine serum albumin (BSA). In separate sets of experiments, cells were treated with a specific HDAC2 inhibitor, MI192 (1–2 μM) or HAT p300 activator, CTPB (10 μM) for 24 h.

**Transient transfection:** Caco-2 cells were transfected with the p300 mammalian expression vector (GenScript, Piscataway, NJ, USA) by electroporation using the Amaxa Nucleofector System as described previously [11,55]. Briefly, ~1 × 10^6^ Caco2 cells were harvested and then electroporated in 100 μL of nucleofector solution T (catalog#VCA-1002, Lonza Biosciences, Houston, TX, USA) with 30 μg of p300 expression vector per 24-well plate. Cells without the p300 expression vector were transfected with an equal amount of empty vector (pcDNA3.1). 48 h post-transfection, cells were washed with PBS and lysed using RNA lysis buffer from Qiagen (Germantown, MD, USA). Human NHE3 or p300 mRNA levels normalized to GAPDH (internal control) using primers specific to NHE3 or p300 (Table 2) were assessed by real-time qPCR as previously described [8,11].

**siRNA Transfection:** Caco-2 cells (1 × 10^5^ cells/well) were plated on 6-well Transwell inserts 24 h before transfection. After 24 h, Caco-2 cells were transfected with HDAC2 siRNA (100 nM, Qiagen) or scrambled siRNA (100 nM; negative control, Qiagen) using Lipofectamine 2000 transfection reagent (catalog#11668019, ThermoFisher Scientific, Waltham, MA, USA) as described previously [8,11]. Cells were harvested 72 h post-transfection. Real-time qPCR was used to assess the relative levels of human NHE3 mRNA, which were normalized to GAPDH as described above.

**ChIP (Chromatin Immunoprecipitation):** ChIP assays were performed utilizing the commercially available CHIP One-Day Kit (catalog#334481) according to the manufacturer’s instructions (Qiagen, Germantown, MD, USA) as previously described [55]. Briefly, untreated or valproic acid (VPA, 2 mM)-treated confluent (12–14 days) Caco2 cells (grown in 6-well plastic supports) or Caco2 cells transfected with 20 μg of p300 expression vector alone or empty vector (pcDNA3.1) were cross-linked with 1% formaldehyde and chromatin was sonicated, then immunoprecipitated with 5 μg of H3K9/14Ac or H4K12Ac (overnight) at room temperature (Santa Cruz Biotechnology, Dallas, TX, USA). Normal rabbit IgG was used as a control (Santa Cruz Biotechnology, Dallas, TX, USA). After reverse cross-linking and DNA extraction, immunoprecipitated DNA was used as the template for real-time qPCR utilizing primers (1–6) (Table 2) spanning the full length of the NHE3 promoter region (-1509/+127).

**Chromatin accessibility assays:** Chromatin accessibility assays were performed to determine the analysis of chromatin accessibility in gene promoters utilizing the commercially available EpiQuik™ Chromatin Accessibility Assay Kit (catalog#P-1047-48) according to the manufacturer’s instructions (Epigentek, Farmingdale, NY, USA). Briefly, in this assay, chromatin is isolated from control or VPA-treated Caco2 cells and then digested with a nuclease mix. DNA was then isolated and used as the template for real-time qPCR utilizing primers (1–6) (Table 2) spanning the full length of the NHE3 promoter region (-1509/+127). Chromatin states can be identified by the accessibility of DNA to nucleases. Additionally, internal control primers are included in the kit as references for assessing the extent of chromatin accessibility at a specified gene target in the sample DNA and for confirming that the proper enzymatic digestions are achieved. Data was analyzed as fold enrichment (FE), which was calculated by simply using a ratio of amplification efficiency of the (nuclease mix) Nse-treated DNA sample over that of the No-Nse sample. *FE* = 2^(^*^Nse CT^* ^−^ *^no-Nse CT^*^)^ × 100%. FE% > 1600% would indicate that the DNA samples (control or VPA-treated) are properly digested (DNA unavailable for PCR with no significant difference in CT values between digested and undigested samples), and the targeted gene region is in the open chromatin state (euchromatin). FE% < 1600% would indicate that the DNA samples (control or VPA-treated) are partially digested (DNA available for PCR with significant difference in CT values between digested and undigested samples) and the targeted gene region is in the partially open chromatin state.

**In vivo studies:** Animal studies were conducted with prior approval of the Animal Care Committee of the University of Illinois Chicago and Jesse Brown Veteran Affairs Medical Center, Chicago, IL, USA. 8-wk-old male C57BL/6J mice were obtained from Jackson Laboratory (Bar Harbor, ME, USA). Mice were acclimatized for 7 days and were given drinking water and standard rodent pellets ad libitum. Mice were divided into two groups: (1) Untreated (control) group (1× PBS) and (2) VPA-treated group (150 or 400 mg/kg body wt., i.p., for 3 days) as previously described [20]. Both of these VPA doses have been shown to be equivalent to a physiological/therapeutic dose in humans [56]. After 3 days, mice were sacrificed, and the ileum and colon were harvested. Mucosa from the ileum and colon were scraped for the preparation of RNA and protein. For immunofluorescence studies, proximal colonic tissues from untreated (control) or VPA-treated mice were frozen in OCT medium (Sakura Finetek, Torrance, CA, USA), and 5 μm sections were cut using a cryostat (Sakura Finetek, Torrance, CA, USA) [11].

### Generation of Mouse/Human Ileal Organoids

Mouse intestinal crypts were isolated, and enteroids were cultured as previously described [57]. Briefly, the small intestine was dissected and opened longitudinally and cut into small (approximately 1 cm) pieces after cleaning with ice-cold 1× PBS containing penicillin, 100 IU/mL/streptomycin, 100 mg/mL) The tissues were rocked in PBS with 2 mM EDTA for 30 min at 4 °C, then transferred to PBS with 54.9 mM D-sorbitol and 43.4 mM sucrose. The tissues were then vortexed for 1–2 min and filtered through a 70-mm sterile cell strainer. The crypts were collected by centrifugation, and ~500 crypts were suspended in 50 mL growth factor-reduced phenol-free Matrigel (catalog#356255, BD Biosciences, San Jose, CA, USA). Next, a 50-mL droplet of Matrigel/crypt mix was placed in the center of the well of a 12-well plate. After 30 min of polymerization, 650 mL of IntestiCult™ Organoid Growth Medium (mouse) (catalog#06005, STEMCELL Technologies, Cambridge, MA, USA) was added to the culture. The medium was changed every 2–3 days. For passage, enteroids were removed from Matrigel, mechanically disrupted by passing through a 27-gauge needle (BD Biosciences), and then transferred to fresh Matrigel. The passage was performed every 7–10 days with a 1:4 split ratio. Human ileal enteroids were generated from pinch biopsies in a similar manner to that mentioned above. Human ileal enteroids were grown in 8-well chamber slides for immunostaining studies as previously described [57]. Prior to RNA extraction or NHE3 immunostaining, mouse or human ileal enteroids were treated with HDAC2 inhibitor, MI192 (2 or 5 μM) for 24 h.

**Real-time Quantitative PCR:** Total RNA was prepared from control (untreated) or VPA-treated Caco2 cells and mouse ileal or proximal colonic mucosal samples, or untreated and MI192-treated mouse ileal enteroids or empty vector (pcDNA3.1) or p300-transfected Caco2 cells utilizing RNeasy mini kit (Qiagen, Germantown, MD, USA) according to the manufacturer’s instructions. cDNA synthesis and PCR amplification were carried out with the SYBR Green one-step real-time PCR master mix using the Mx3000p machine (Agilent Technologies, Santa Clara, CA, USA). Human or mouse NHE3 and GAPDH were amplified with gene-specific primers (Table 2) as previously described [8,11]. The quantification was expressed as a ratio of 2^Δ^Ct^-NHE3^/2^Δ^Ct^-GAPDH^, where ΔCt-NHE3 and ΔCt-GAPDH represent the difference between the threshold cycle of amplification of treated and control RNA for NHE3 and GAPDH, respectively [8,11].

**Cell lysates and Western blotting:** Lysates were prepared from control or treated Caco2 cells or ileal and proximal colonic mucosa as previously described [8,11,55]. Lysates were run on a 7.5% gel and then transferred onto a nitrocellulose membrane. Immunoblotting was carried out using an NHE3 antibody as previously described [8,11]. Bands were visualized with enhanced chemiluminescence (ECL) detection reagents.

**Immunofluorescence staining:** Proximal colonic tissue of untreated or VPA-treated mice was sectioned at 5 μm thickness onto glass slides and fixed with 1% paraformaldehyde as previously described [11,57,58]. Proximal colonic sections or human ileal enteroids grown in chamber slides were stained utilizing primary antibodies (1:100): rabbit anti-NHE3 (a generous gift from Dr. Chris Yun, Emory University, Atlanta, GA, USA) or monoclonal anti-mouse villin (Santa Cruz Biotechnology) and anti-goat secondary antibodies conjugated to either anti-rabbit Alexa fluor 488 (green) or anti-mouse Alexa fluor 568 (red) (ThermoFisher Scientific) or Phalloidin (Invitrogen, Waltham, MA, USA) as previously described [57]. The slides were mounted with Prolong Gold Antifade-DAPI (ThermoFisher Scientific). Stained sections were imaged using a Zeiss Axio Observer 5 inverted fluorescence microscope (Zeiss, White Plains, New York, NY, USA) equipped with an Axiocam 702 camera at 20× magnification (Zeiss, White Plains, New York, NY, USA). Stained human ileal enteroids were imaged using a Zeiss LSM 710 BiG confocal microscope (Zeiss, White Plains, New York, NY, USA) equipped with a Plan-Apochromat 20× objective (Zeiss, White Plains, New York, NY, USA) and controlled by ZEN 2.3 software.

**HiChIP-seq and data analysis:** Epithelial cells were isolated from the ileum of untreated control or VPA-treated mice. To obtain an adequate number of epithelial cells (20 × 10^6^), IECs were isolated from the ileum of untreated controls (3 mice pooled as one sample) or VPA-treated mice (4 mice pooled as one sample). The HiChIP protocol was performed in accordance with the guidelines provided by Dovetail Genomics (Scotts Valley, CA, USA). Cells underwent cross-linking, followed by MNase fragmentation, to eliminate the need for sonication and achieve nucleosome-level resolution. Subsequently, chromatin fragments were immunoprecipitated using an H3K27Ac antibody. Following chromatin immunoprecipitation (ChIP), optimized proximity ligation was performed to efficiently convert free ends into ligation products, followed by effective cross-link reversal with biotin enrichment to minimize loss of target molecules. Standard paired-end sequencing was performed on an Illumina NGS instrument(Ilumina, San Diego, CA, USA). The HiChIP sequencing yielded approximately 224 million total read pairs, exhibiting a 2% PCR duplicate rate, 80% valid read pairs, and a signal-to-noise ratio of 2.5. Coverage profiles for H3K27ac were generated for both VPA-treated (blue) and untreated control (green) samples across a 122-kb genomic region encompassing the Slc9a3 gene located on chromosome 13. Sequencing alignments (align reads to the mouse (mm9) genome assembly) were converted into bedGraph coverage tracks using deepTools bamCoverage [59,60]. (https://deeptools.readthedocs.io/en/develop/content/tools/bamCoverage.html). Upper-triangular sparse contact matrices were extracted from mcool files at a 4-kb resolution utilizing cooler dump. Subsequently, coverage profiles and chromatin contacts were visualized in ‘R’ version 4.6.1 through the Bioconductor package, Sushi [61]. Contact frequency was defined as the number of HiChIP read pairs linking two genomic loci. Contacts exhibiting frequencies that exceeded the sample-specific mean contact frequency plus one standard deviation (mean + 1 SD) were retained and illustrated as arcs. This criterion served as a visualization threshold for higher-frequency contacts and does not signify a formal statistical significance test of individual contacts or differential interactions between VPA-treated and control samples. The height of the arcs is scaled according to the genomic distance between the interacting loci and does not reflect the strength of the interactions. The RefSeq track indicates the genomic positions of Slc9a3 and its neighboring genes.

**Statistical analysis:** Data are presented as mean ± SEM. Normality of the data was assessed using the Shapiro–Wilk test before applying parametric statistical analyses. Comparisons between two groups were performed using an unpaired two-tailed Student’s *t*-test. Comparisons among multiple groups were analyzed by one-way ANOVA, followed by Dunnett’s test. *p* < 0.05 or less was considered statistically significant. Statistical analyses were performed using GraphPad Prism version 11.

## 5. Conclusions

Our results reveal novel regulatory mechanisms that enhance intestinal NHE3 gene expression through histone acetylation-related epigenetic modifications. While VPA affects multiple class I and IIa HDACs, specifically HDACs 1, 2, 3, 4, 5, 7, 8, and 9, our follow-up experiments identified HDAC2 as a key regulator of intestinal NHE3 expression. This conclusion was reinforced by additional pharmacological and genetic methods. Targeted inhibition of HDAC2/3 using MI192 notably enhanced NHE3 expression in intestinal epithelial cells as well as in mouse and human ileal enteroids, mirroring the effects seen with VPA. Notably, the siRNA-mediated knockdown of HDAC2 also led to a considerable increase in NHE3 expression, providing independent validation that the observed effect was not solely attributable to pharmacological inhibition. These results underscore the functional relevance of HDAC2 inhibition in regulating intestinal NHE3 expression. These novel mechanisms of NHE3 upregulation may lay the groundwork for innovative, more effective treatment options for intestinal disorders such as IBD-associated diarrhea, in which NHE3 expression/function is downregulated.

## Figures and Tables

**Table 1:** Materials used for Experimental Studies.

Chemicals/Reagents	Company	Catalog Number
Valproic Acid (VPA)	Millipore Sigma (Burlington, MA, USA)	P4543
Trichostatin A (TSA)	Millipore Sigma	T8552
CTPB (p300 activator)	Millipore Sigma	SML0694
MI192 (HDAC2/3 inhibitor)	Tocris Biosciences (Bristol, UK)	5647
ProLong Gold Antifade DAPI	ThermoFisher Scientific	P36935
Antibodies		
NHE3 (rabbit polyclonal antibody)	Generous gift from Dr. Chris Yun, Emory University, Atlanta, GA, USA	
Acetylated Histone 3 (H3K9/K14 Ac) (rabbit polyclonal antibody)	Santa Cruz Biotechnology	sc-8655-R
Acetylated Histone 4 (H4K12 Ac) (rabbit polyclonal antibody)	Santa Cruz Biotechnology	sc-8661-R
Normal Rabbit IgG antibody	Santa Cruz Biotechnology	sc-2027
Villin (mouse monoclonal antibody)	Santa Cruz Biotechnology	sc-58897
GAPDH (rabbit monoclonal antibody)	ThermoFisher Scientific	MA5-35235
Goat anti-mouse Rhodamine Phalloidin	ThermoFisher Scientific	R-6393
Goat anti-mouse HRP (secondary antibody)	Santa Cruz Biotechnology	sc-2005
Goat anti-rabbit HRP (secondary antibody)	Santa Cruz Biotechnology	sc-2004
Goat anti-rabbit Alexa Fluor 488 (secondary antibody)	ThermoFisher Scientific	A-11008
Goat anti-mouse Alexa Fluor 568 (secondary antibody)	ThermoFisher Scientific	A-11004

**Table 2:** Gene-specific Primer Sequence used for Real-time qPCR, ChIP, and Chromatin Accessibility Assays.

Gene	Forward 5′-3′	Reverse 5′-3′
**Real time qPCR**		
Human NHE3	ACCTGTTCGTCAGCACCAC	GCTCGCTCCTCTTCACCTT
Human GAPDH	GAAATCCCATCACCATCTT	AAATGAGCCCCAGCCTTCT
Mouse NHE3	GGCCTTCATTCGCTCCCCAAG	ATGCTTGTACTCCTGCCGAGG
Mouse GAPDH	TGTGTCCGTCGTGGATCTGA	CCTGCTTCACCACCTCTTGAT
**ChIP & Chromatin Accessibility**		
**(NHE3 Promoter)**		
Primer 1	AGCAAGACATTCCCAGGGTGAG	GTTTGGACGGCTCTTGTGTGC
Primer 2	GCACACAAGAGCCGTCCAAAC	CCTTCAGTCCCTCCTATTGG
Primer 3	CCAATAGGAGGGACTGAAGG	CTCAGGCTCCACTGTGCAAA
Primer 4	AGAGAGACTTACCAGCGCAC	CCAACCCTCCTTCATAAACGC
Primer 5	TGCACCCTTGGGCCGCCCTG	GCTCCCTGGTGCCCACCCTC
Primer 6	GGGAGCTGCGGCGGTGTCTG	CCACATTGCCGCCTGCTCAGC

## Data Availability

The original contributions presented in this study are included in the article. Further inquiries can be directed to the corresponding author.

## Supplementary Materials

The following supporting information can be downloaded at: https://www.mdpi.com/article/10.3390/ijms27188096/s1.

## Abbreviations

The following abbreviations are used in this manuscript:

5′-Aza	5′-Azacytidine
Bp	Base pair
BSA	bovine serum albumin
CD	Crohn’s disease
cDNA	Complementary DNA
ChIP	Chromatin immunoprecipitation
CpG	Cytosine-phosphate-Guanine
CGI	CpG island
Ct	Cycle threshold
CSD	Congenital sodium diarrhea
DMT	DNA methyl transferase
DNA	Deoxyribonucleic acid
DSS	Dextran sodium sulphate
ECL	Enhanced chemiluminescence
EV	Empty vector
FBS	Fetal bovine serum
FE	Fold enrichment
GAPDH	Glyceraldehyde phosphate dehydrogenase
H^+^	Hydrogen
HAT	Histone acetylase
H3	Histone 3
H3K27Ac	Acetylation at lysine residue 27 of Histone 3
H4	Histone 4
HDAC	Histone deacetylase
HNF4α	Hepatocyte nuclear factor 4α
IBD	Inflammatory Bowel Diseases
IECs	Intestinal epithelial cells
IgG	Immunoglobulin G
i.p.	Intraperitoneal
kb	Kilobase
K9/14	Lysine residue 9 or 14 on histone 3
K12	Lysine residue 12 on histone 4
MBD	Methyl CpG binding proteins
miRNA	MicroRNA
MNase	Micrococcal nuclease
mRNA	Messenger RNA
Na^+^	Sodium
NHE	Sodium-hydrogen exchanger
OCT	Optimal cutting temperature
p300	E1A binding protein 300
PBS	Phosphate-buffered saline
pcDNA	Plasmid cloning DNA
PKA	Protein kinase A
PP1	Protein phosphatase 1
PP2A	Protein phosphatase 2A
qPCR	Quantitative polymerase chain reaction
RNA	Ribonucleic acid
SAHA	Suberoylanilide hydroxamic acid
siRNA	Small interfering RNA
Sp1	Specificity protein 1
Sp3	Specificity protein 3
SLC9A3	Solute carrier family9 member A3
TNBS	2,4,6-trinitrobenzenesulfonic acid
TNFα	Tumor necrosis factor α
TSA	Trichostatin A
UC	Ulcerative colitis
VPA	Valproic acid
