# Supplementary material for: A Novel Epigenetic Mechanism of Intestinal NHE3 Gene Regulation by Histone Acetylation
Source: Int J Mol Sci. 2026 Sep 11;27(18):8096. doi: 10.3390/ijms27188096 (PMC13607783; doi:10.3390/ijms27188096)
Supplement: Supplementary file 1 [file ijms-27-08096-s001.zip › ijms-4469303-supplementary.pdf]

Supplementary Figure S1

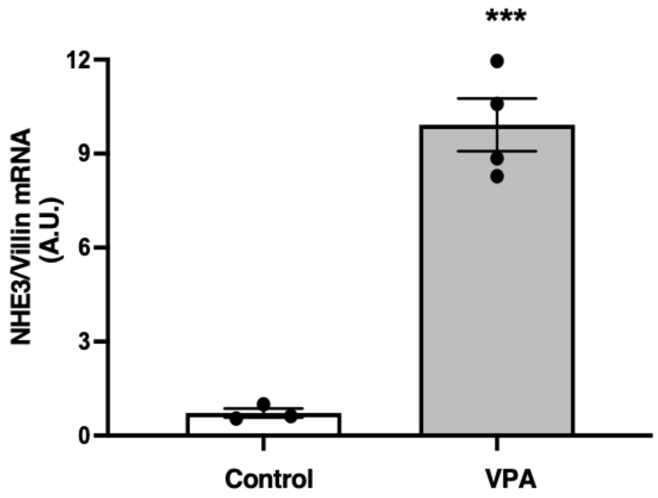

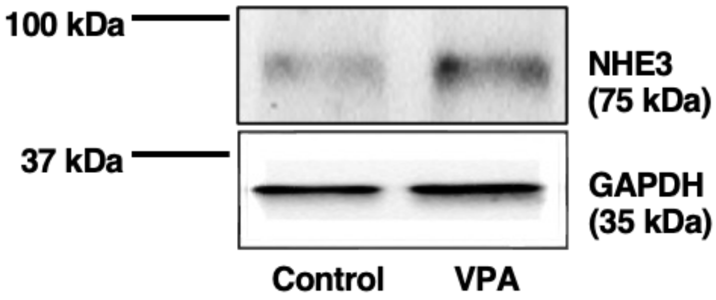

Valproic acid increases NHE3 expression in intestinal epithelial cells isolated from mouse ileum: VPA (400 mg/kg body wt., i.p.) was administered to mice once daily for 3 days. Total RNA and cell lysates were then extracted from the ileal IECs. NHE3 mRNA and protein expression was assessed by real time qPCR and western blot (representative blot is shown) respectively. qPCR data are expressed as fold increase in arbitrary units compared with untreated control ((set as 1 for mRNA). Results represent means ± SEM of 3-4 mice/group. ***p<0.001 compared with untreated mice (control). Representative western blot shows untreated control (IECs isolated from the ileum of 3 mice pooled as one sample) or VPA-treated (IECs isolated from the ileum of 4 mice pooled as one sample).
